# Structural and functional analysis of Ccr1l1, a Rodentia-restricted eosinophil-selective chemokine receptor homologue

**DOI:** 10.1016/j.jbc.2021.100373

**Published:** 2021-02-03

**Authors:** Jaclyn M. Kline, Lauren E. Heusinkveld, Eleanor Taranto, Clare B. Martin, Alessandra G. Tomasi, Isabel J. Hsu, Kyoungin Cho, Jaspal S. Khillan, Philip M. Murphy, Sergio M. Pontejo

**Affiliations:** 1 Laboratory of Molecular Immunology, National Institute of Allergy and Infectious Diseases, National Institutes of Health, Bethesda, Maryland, USA; 2 Comparative Medicine Branch, National Institute of Allergy and Infectious Diseases, National Institutes of Health, Bethesda, Maryland, USA

**Keywords:** chemotaxis, cell migration, calcium flux, receptor internalization, Ccr1, eosinophil, tyrosine sulfation, sulfotyrosine, constitutive activity, 7TM, 7 transmembrane domain, ACKR, atypical chemokine receptor, BMDE, bone marrow-derived eosinophils, BMDM, bone marrow-derived macrophages, BRET, bioluminescence resonance energy transfer, Ccr1l1, Ccr1-like 1, ECL, extracellular loop, FACS, fluorescence-activated cell sorting, GPCR, G protein-coupled receptor, ICL, intracellular loop, PBS, phosphate-buffered saline, TM, transmembrane domain

## Abstract

Mouse *Ccr1l1* (Ccr1-like 1) encodes an orphan G-protein-coupled receptor (GPCR) with the highest homology to the inflammatory and highly promiscuous chemokine receptors Ccr1 and Ccr3 (70 and 50% amino acid identity, respectively). *Ccr1l1* was first cloned in 1995, yet current knowledge of this putative chemokine receptor is limited to its gene organization and chromosomal localization. Here we report that Ccr1l1 is a Rodentia-specific gene selectively expressed in eosinophils. However, eosinophil phenotypes, development, and responsiveness to chemokines were all normal in naïve Ccr1l1 knockout mice. We demonstrate for the first time that recombinant Ccr1l1 is expressed on the plasma membrane of transfected cells and contains an extracellular N terminus and an intracellular C terminus, consistent with GPCR topology. Using receptor internalization, β-arrestin recruitment, calcium flux, and chemotaxis assays, we excluded all 37 available mouse chemokines, including Ccr1 ligands, and two viral chemokines as Ccr1l1 ligands, and demonstrated that mouse Ccr1, but not Ccr1l1, exhibits constitutive signaling activity. However, sequence analysis and structural modeling revealed that Ccr1l1 is well equipped to act as a classical signaling GPCR, with N-terminal sulfotyrosines as the only signaling and chemokine-binding determinant absent in Ccr1l1. Hereof, we show that a sulfatable N-terminal Ccr1 Y^18^ residue is essential for chemotaxis and calcium responses induced by Ccl3 and Ccl9/10, but substituting the corresponding Ccr1l1 F^19^ residue with tyrosine failed to confer responsiveness to Ccr1 ligands. Although Ccr1l1 remains an extreme outlier in the chemokine receptor family, our study supports that it might respond to unidentified mouse chemokine ligands in eosinophil-driven immune responses.

Chemokines are a large family of chemotactic cytokines that regulate immune cell development and migration by signaling through 7-transmembrane domain (7TM) receptors. Chemokines are divided into four subfamilies, termed CC, CXC, XC, and CX3C, based on the number and arrangement of their N-terminal cysteine residues (1). Two types of cellular receptors mediate chemokine functions, class A G-protein-coupled receptors (GPCRs, n = 18) and atypical chemokine receptors (ACKRs, n = 4), which do not signal through G proteins (2, 3, 4). Chemokine GPCRs are classified according to the subfamily of chemokines they bind. Chemokine receptors constitute one of the largest subfamilies of the 7TM receptor superfamily.

Chemokine receptors are integral plasma membrane proteins, consisting of an extracellular N terminus, an intracellular C terminus, and 7 transmembrane domains (TMI–VII) connected by three intracellular loops (ICL) and three extracellular loops (ECL) (5). In addition, two disulfide bridges, one common to most class A GPCRs between TMIII and ECL2, and the other more specific for chemokine GPCRs between TMVII and the N terminus, maintain the folding of essential ligand binding and activation determinants of the receptor (6, 7, 8, 9, 10, 11). Based on the classical “two-step/two-site” model, the ligand binding and signaling determinants of chemokine receptors are functionally and spatially separated in the N-terminus and the TM domains, respectively (12, 13, 14, 15). This model proposes that while the N terminus is mainly responsible for the affinity of the interaction by its binding to the globular core of the chemokine, the TM domains initiate conformational changes upon binding of the unstructured N-terminal domain of the chemokine, which ultimately trigger intracellular signaling. However, recent detailed structural studies, including the crystallographic structure of the first chemokine-receptor complexes, describe a higher degree of overlap in the regulation of chemokine binding and activation by more extended areas of the receptor (16, 17, 18, 19, 20). In addition, ligand binding and selectivity of chemokine receptors are known to be further regulated by posttranslationally modified sulfated tyrosines in the N-terminal domain (21, 22).

Chemokine GPCR intracellular signaling is transduced by heterotrimeric G proteins and/or β-arrestins, resulting in the release of intracellular stores of ionized calcium, actin polymerization and cell migration, or receptor internalization, respectively (23, 24). After chemokine binding, G protein subunits dissociate from the receptor to activate various signaling pathways allowing β-arrestin recruitment and the subsequent internalization of the receptor (25). An Asp-Arg-Tyr (DRY) motif at the boundary between TMIII and ICL2 of the receptor is known to be an essential G-protein-coupling site in GPCRs (26, 27). GPCR mutants for the DRY motif typically lose the ability to activate G proteins but retain β-arrestin-mediated signaling (28, 29). Similarly, the substantial divergence of the DRY motif of ACKRs from the canonical DRYLAIV sequence is thought to partially explain the β-arrestin-biased signaling of these chemokine scavenger receptors (4). Although unable to induce cell migration, ACKR-mediated chemokine internalization is essential for regulating chemotactic gradients and chemokine availability during immune and inflammatory responses (30). In addition, some chemokine receptors constitutively induce G-protein-dependent signaling and/or β-arrestin-dependent internalization without the need for chemokine binding (31, 32). This ligand-independent activity is particularly common among virally encoded chemokine receptors (33). Furthermore, adding to the complexity, some receptors are known to interfere with the signaling of other chemokine receptors by the formation of heterodimers (34, 35, 36, 37).

Chemokine receptors can also be classified as homeostatic or inflammatory depending on their functional roles (2, 38). Most of the 14 known inflammatory chemokine receptor subtypes bind distinct subsets of multiple inflammatory chemokines, and for closely related receptors, the subsets typically overlap (2). For instance, Ccr1 and Ccr3 have six (Ccl3, Ccl4, Ccl5, Ccl6, Ccl7, and Ccl9/10) and five (Ccl5, Ccl7, Ccl9/10, Ccl11, and Ccl24) known chemokine ligands, respectively, three of which are the same. Ccr1l1 (Ccr1-like 1) is a putative 7TM mouse protein identified by gene cloning in 1995 (39). There have been no subsequent peer-reviewed papers in the ensuing 25 years characterizing Ccr1l1; however, bioinformatics databases contain evidence that it lacks a human orthologue and is the only gene located between Ccr1 and Ccr3 on mouse chromosome 9. In the ImmGen database, Ccr1, Ccr3, and Ccr1l1 mRNA are all listed as present in mouse eosinophils and some macrophages, with the highest Ccr1l1 expression in eosinophils. Ccr1l1 has 70% amino acid identity to mouse Ccr1 and 50% amino acid identity to mouse Ccr3, yet Ccr1l1 ligands have never been reported, nor has it been established that Ccr1l1 can be expressed on the plasma membrane. Here, we addressed these questions by a systematic and broad structural and functional interrogation of Ccr1l1.

## Results

### Ccr1l1 is a Rodentia-restricted putative chemokine receptor highly related to mouse Ccr1

**Figure 1:**
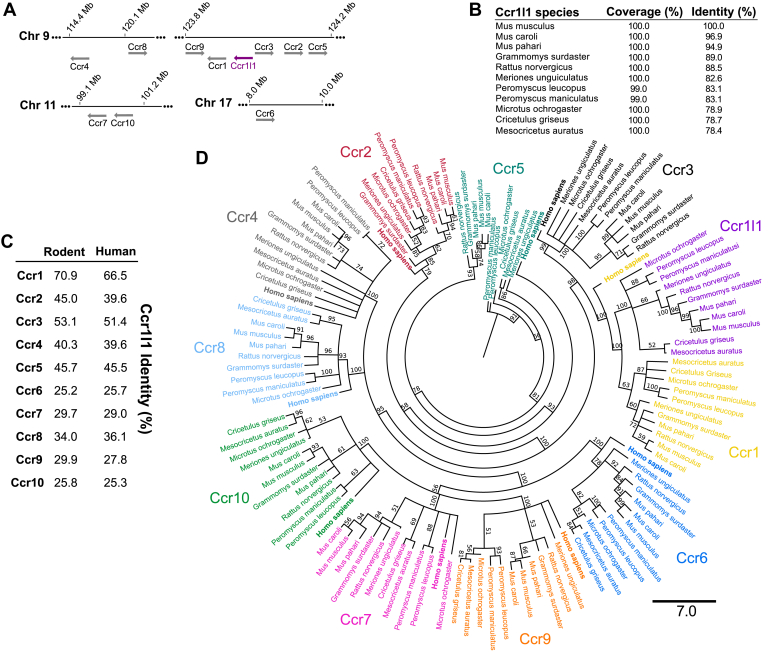
**Ccr1l1 is highly conserved in rodents and closely related to Ccr1**. *A*, schematic representation of the chromosome localization of mouse CC chemokine receptor genes in the mouse genome. *Solid horizontal lines* represent chromosome 9 (Chr 9), Chr 11, and Chr 17 nucleotide sequences. Reference nucleotide positions are numbered above each chromosome line. Distances are not at proportional scale. *Arrows* indicate the ORF and transcription orientation for each gene. The Ccr1l1 locus is depicted in *purple*. *B*, amino acid sequence identity in % of mouse Ccr1l1 with 11 rodent orthologues identified by BLAST-P. *C*, amino acid sequence identity in % of Ccr1l1 with rodent and human CC chemokine receptors. The average identity of 11 rodent Ccr1l1 species with the human or all the rodent orthologues of each receptor is indicated. *D*, maximum likelihood phylogenetic tree of the amino acid sequences for CC chemokine receptors (color-coded and labeled on the outside of the tree) of human (highlighted in *bold typeface*) and the 11 rodent species listed in panel *C*. Sequences were aligned using MUSCLE and analyzed by Randomized Axelerated Maximum Likelihood (RAxML). The consensus tree of 1000 bootstrap inferences is shown. The consensus support for each branched group is indicated in %. A scale bar of seven amino acid substitutions per site is shown below the tree.

Given its extremely strong homology with Ccr1 and Ccr3, we framed our initial analyses of Ccr1l1 within the CC chemokine receptor subfamily. Ccr1l1 is located on mouse chromosome 9, together with seven other CC chemokine receptors (Ccr1, Ccr2, Ccr3, Ccr4, Ccr5, Ccr8, and Ccr9) (Fig. 1*A*). Ccr1l1 maps to chromosomal region 9qF4 flanked by Ccr1 and Ccr3. This region is syntenic to human 3p21, which contains CCR1 and CCR3 but lacks a Ccr1l1 ortholog. Ccr1 and Ccr1l1 are separated by approximately 14 kb and have the same transcription orientation (Fig. 1*A*).

A BLAST-P search using the predicted mouse full-length Ccr1l1 protein sequence as the query revealed orthologs in at least ten other rodent species with amino acid identities >78% (Fig. 1*B*). Notably, no Ccr1l1 orthologs were found in any other mammalian families, indicating that Ccr1l1 is a highly conserved gene restricted to Rodentia. Ccr1l1 amino acid sequence is 70% and 53% identical to mouse Ccr1 and Ccr3, respectively, whereas it has slightly lower amino acid identity to human CCR1 (66%) and CCR3 (51%), and substantially lower homology with other CC chemokine receptors (Fig. 1*C*). To understand the evolutionary relationship of Ccr1l1 with other CC chemokine receptors, we performed a phylogenetic analysis of all CC chemokine receptors found in these 11 rodent species and humans. The different chemokine receptors were correctly clustered by a maximum likelihood analysis that established evolutionary relationships—common ancestors shared by Ccr6, Ccr7, Ccr9 and Ccr10; by Ccr4 and Ccr8; and by Ccr2 and Ccr5—similar to a previously published phylogenetic analysis (Fig. 1*D*) (2). We found that Ccr1l1 shares a common ancestor with Ccr1 and Ccr3 and appears to have evolved directly from Ccr1 (Fig. 1*D*). In conclusion, mouse Ccr1l1 and Ccr1 are two highly related receptors with similar chromosomal location, transcription orientation, amino acid sequence, and evolution.

### Ccr1l1 is selectively expressed by macrophages and eosinophils

**Figure 2:**
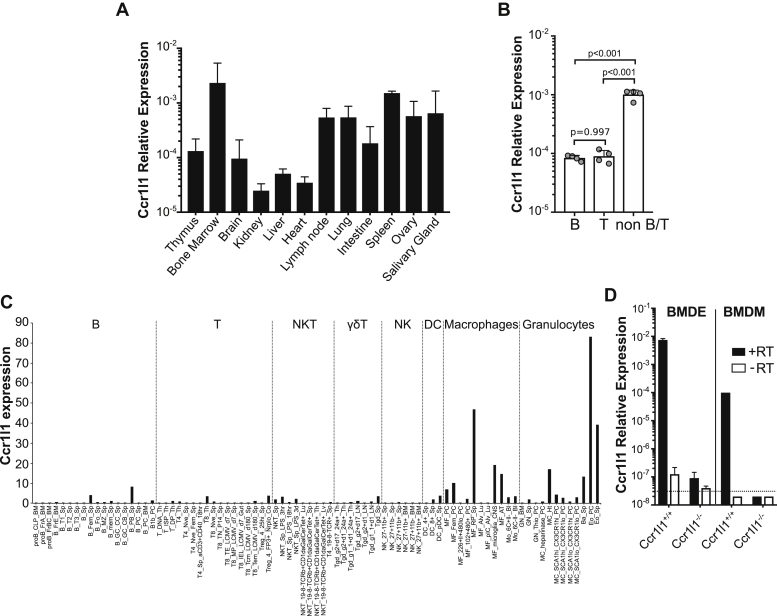
**Ccr1l1 is primarily expressed in eosinophils**. *A*, tissue expression. Ccr1l1 expression in the tissues indicated on the *x*-axis from C57BL/6NTac mice detected by qPCR. Bars represent the mean ± SD relative abundance of Ccr1l1 mRNA from three mice. Data are from one experiment representative of four independent experiments. *B*, splenocyte expression. The relative abundance of Ccr1l1 transcripts was analyzed by qPCR in B cells (B), T cells (T), and non-T/non-B cells (non B/T) sorted from spleens of C57BL/6NTac mice (n = 4). Bars represent the mean ± SD from one experiment representative of three independent experiments. *Gray circles* represent each individual mouse. *p* values from a one-way ANOVA with Tukey correction for multiple comparisons are indicated. *C*, single cell expression analysis. Single cell RNA-Seq results compiled by ImmGen database reporting Ccr1l1 expression in various murine leukocyte populations (*x*-axis). Data are split by vertical *dashed lines* into different leukocyte populations labeled above the graph. *D*, Ccr1l1 expression in bone-marrow-derived eosinophils (BMDE) and macrophages (BMDM). The cells indicated above the graph were derived from bone marrow obtained from *Ccr1l1*^*+/+*^ or *Ccr1l1*^*−/−*^ mice (*x*-axis). Bars represent the mean ± SD relative Ccr1l1 expression of quadruplicates of retrotranscribed samples (+RT, *black bars*) or nonretrotranscribed controls (−RT, *white bars*). Data are from one experiment representative of three independent experiments.

Next, we investigated the tissue and cellular expression of Ccr1l1. In the absence of an available antibody reagent to detect Ccr1l1 protein, we analyzed Ccr1l1 mRNA expression in multiple tissues and cell types by qPCR. Relative transcript abundance of Ccr1l1 was particularly high in the bone marrow and the spleen of naïve C57BL/6NTac mice compared with other tissues such as the brain, kidney, liver, or heart (Fig. 2*A*). Then, we sorted naïve splenocytes into T, B, and non-T/non-B cell (non B/T) populations and found that Ccr1l1 expression was significantly higher in non-B/T cells (Fig. 2*B*). Consistent with this, a search in the RNA-seq Gene Skyline databrowser of the Immunological Genome Project or ImmGen (http://rstats.immgen.org/Skyline/skyline.html) (40) revealed that Ccr1l1 expression is mostly restricted to macrophages and eosinophils (Fig. 2*C*). To confirm this, we analyzed the expression of Ccr1l1 in bone-marrow-derived macrophages (BMDM) and eosinophils (BMDE) generated *in vitro* from wild-type *Ccr1l1*^*+/+*^ C57BL/6NTac mice or *Ccr1l1*^*−/−*^ mice as a control. We found that while both BMDM and BMDE expressed Ccr1l1, the relative expression was 2 logs higher in BMDE (Fig. 2*D*). As expected, Ccr1l1 was undetectable or near the detection limit in cells derived from *Ccr1l1*^*−/−*^ animals or in nonretrotranscribed controls (Fig. 2*D*). We concluded that Ccr1l1 is expressed in most lymphoid tissues and in some myeloid cells, with the highest constitutive expression in eosinophils.

### Ccr1l1 contains common structural characteristics of CC chemokine receptors

**Figure 3:**
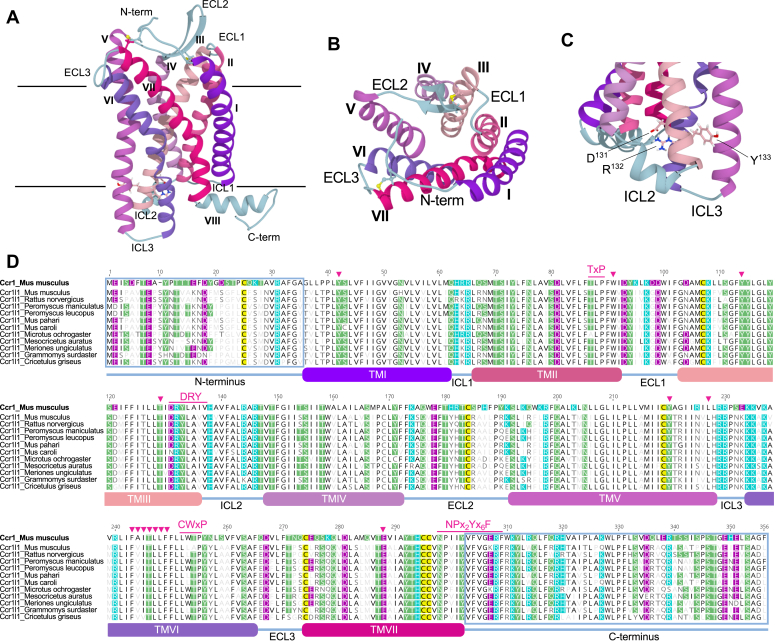
Ccr1l1 preserves all major sequence and structural components of chemokine GPCRs. *A*,. Ccr1l1 structural model generated by I-TASSER. Ccr1l1 is depicted in ribbons with predicted transmembrane α-helices numbered I-VII (TMIVII) and colored in different tones of *purple* and *pink*. N terminus (N-term), C terminus (C-term), and intracellular loops (ICL) and extracellular loops (ECL) are shown in *blue*. *Horizontal black lines* represent the limits of the plasma membrane. *B*, top view of the extracellular side of the Ccr1l1 molecular model. Disulfide bonds between the N terminus and the beginning of the TMVII and between the beta hairpin in ECL2 and the TMIII are shown in *yellow balls* and *sticks*. *C*, Ccr1l1 contains the signature DRY signaling motif of chemokine GPCRs. The side chains of D^131^, R^132^, and Y^133^, colored by heteroatom are shown at the end of TMIII, right before the beginning of ICL2. *D*, amino acid sequence alignment of mouse Ccr1 with 11 Ccr1l1 species. The approximate amino acid sequence of the different loops and transmembrane domains are indicated below the alignment with *lines* and *boxes*, respectively, colored following the same color pattern as in panels (*A–C*). The sequences of the N terminus and the C terminus are framed in blue. Signaling amino acid motifs highly conserved in class A GPCRs (TxP, DRY, CWxP, and NPx2Yx6F (41)) are indicated above the alignment. Similarly, *pink arrowheads* above the sequences point at conserved positions directly involved in the signal transmission and activation of chemokine receptors (42). Conserved acidic, basic, and polar residues with mouse Ccr1 (reference sequence, on top) are shaded in rodent Ccr1l1 sequences in *purple*, *cyan*, and *green*, respectively. Conserved hydrophobic residues are shown in *black letters* in white background. Symbols of nonconserved residues in rodent Ccr1l1 sequences are shown in increasingly *lighter gray* according to the extent of conservation. Cysteines are shaded in *yellow*.

To assess the possibility that Ccr1l1 acts as a canonical GPCR, we next looked in both the primary amino acid sequence and a three-dimensional model of Ccr1l1 for classical structural and signaling features of chemokine GPCRs. A non-template-restricted homology model generated by I-TASSER predicted that Ccr1l1 preserves the 7TM domains, 3 ICL, 3 ECL, and the N-terminal and C-terminal domains that define the topology of chemokine GPCRs (Fig. 3*A*). In addition, this model supported that the β-hairpin fold typically observed in the ECL2 of chemokine receptors and the two conserved extracellular disulfide bridges (TMVII-N-terminus and TMIII-ECL2) are preserved in Ccr1l1 (Fig. 3*B*). Importantly, the model predicted that Ccr1l1 conserves the G-protein-coupling DRY motif at the boundary between TMIII and ICL2 (Fig. 3*C*). Alignment of the amino acid sequences of 11 different rodent Ccr1l1 species with mouse Ccr1 confirmed that Ccr1l1 preserves the canonical DRYLAIV sequence of the DRY motif, with most rodent Ccr1l1 orthologues encoding a DRYLAVV sequence (Fig. 3*D*). This exact sequence is also found in the corresponding location of human CCR8 and CCR5 (41).

Other conserved motifs have been implicated in the activation of class A GPCRs; in particular, TxP in TMII, CWxP in TMVI, and NPx_2_Yx_6_F at the boundary between TMVII and the C terminus (41). As shown in Figure 3*D,* the TxP and the NPx_2_Yx_6_F motifs are highly conserved in all Ccr1l1 species whereas instead of a CWxP motif, mouse Ccr1 and 6 Ccr1l1 species contain LWxP and the other five Ccr1l1 species, including mouse Ccr1l1, present an LLxP motif. However, it is important to note that while the CWxP motif is highly conserved in other class A GPCRs, of these three signaling motifs, CWxP is the most variable in the chemokine receptor family (C, W, and P are conserved in 41%, 71%, and 99% of chemokine receptors, respectively), and it has been proposed to play only a minor role in signaling by chemokine GPCRs (41).

Recently, a comprehensive mutagenesis analysis of CXCR4 revealed other amino acids required for G protein coupling, activation, and signal transmission (42). For instance, besides the DRY motif, this study identified that conformational changes in CXCR4 S^131^, Y^219^, and Y^302^ are critical to form the structural support for the G protein interface and that L^226^ is directly involved in G protein coupling. Y^302^ is part of the NPx_2_Yx_6_F motif conserved in Ccr1l1, Y^219^ and L^226^ are conserved in equivalent positions (220 and 227, respectively) of Ccr1l1 TMV, and S^131^ is substituted for a highly homologous T^129^ in Ccr1 and all Ccr1l1 species (Fig. 3*D*). In addition, Y^45^, W^94^, Y^116^, and E^288^ located at the bottom of the chemokine binding pocket of CXCR4 were shown to be critical signal initiators (42). As shown in the alignment of Figure 3*D*, these four amino acids are conserved in equivalent positions of Ccr1 and Ccr1l1 (Y^42^, W^91^, Y^114^, and E^288^). Finally, we found that a TMVI hydrophobic bridge involved in the signal propagation from the signal initiators on the extracellular side to the intracellular activation determinants of GPCRs (42, 43, 44), is also conserved in TMVI of Ccr1 and all Ccr1l1 species (Fig. 3*D*). Together, these observations support that Ccr1l1 is well equipped to act as a canonical signaling GPCR.

### Ccr1l1 protein is expressed on the cell membrane

**Figure 4:**
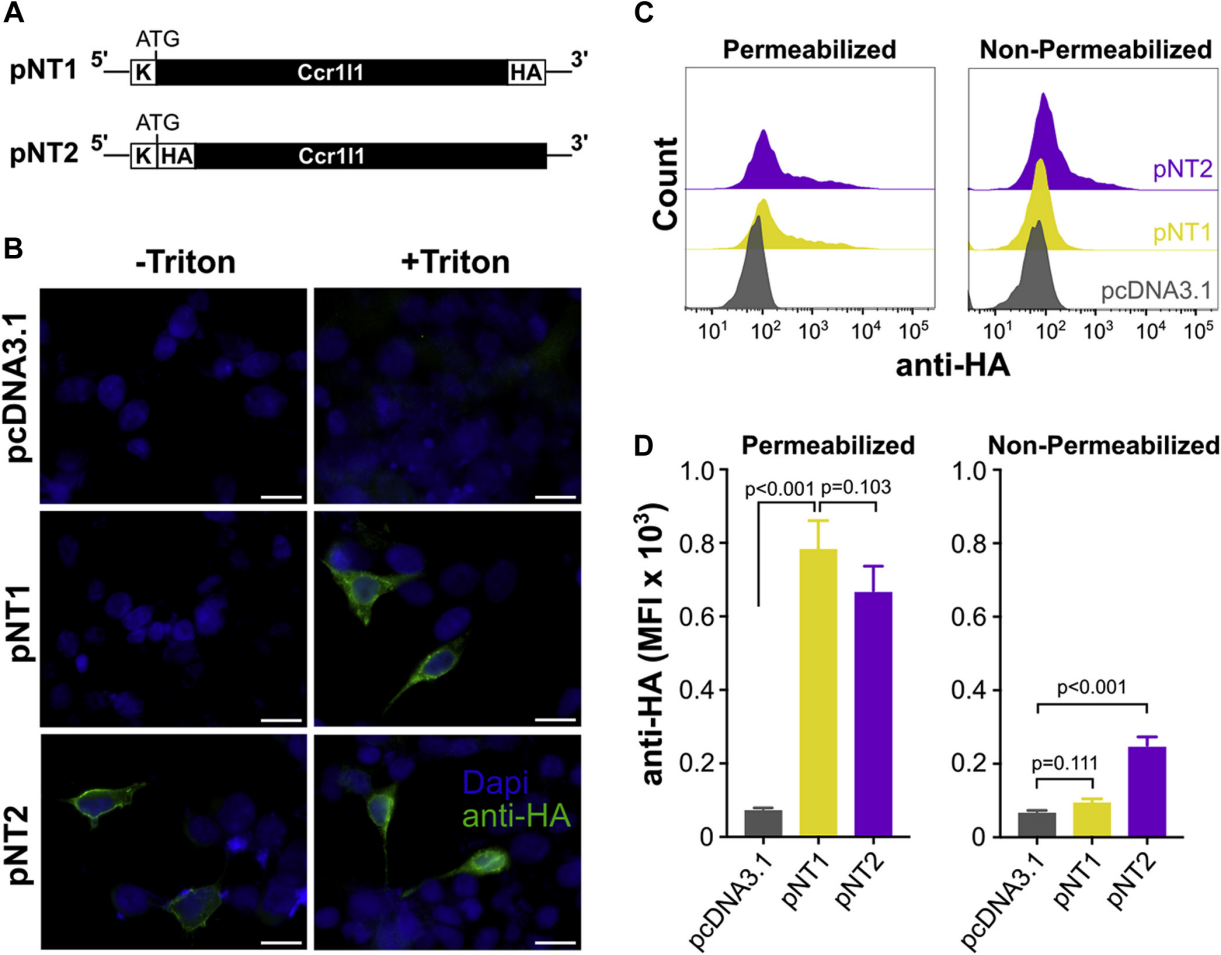
**Ccr1l1 encodes a transmembrane protein that traffics to the plasma membrane and contains an extracellular N terminus and an intracellular C terminus.***A*, schematic representation of the pNT1 and pNT2 constructs cloned into a pcDNA3.1 plasmid for the expression of Ccr1l1 with a C-terminal or N-terminal HA tag, respectively. Ccr1l1 ORF is indicated by a *black box*, whereas the HA tag (HA) and kozak sequence (K) are shown in *white boxes*. The position of the ATG initiation codon is indicated above each graph. The expression of these constructs was analyzed by fluorescence microscopy (*B*) and FACS (*C* and *D*) in HEK293-transfected cells. *B*, fluorescence microscopy images of the anti-HA (*green*) and DAPI (*blue*) staining of HEK293 cells transfected with the plasmids indicated on the left of each row with (+Triton) or without (–Triton) permeabilization, as indicated above each column (400× magnification, scale bar = 20 μm). *C*, FACS histograms of the anti-HA staining of permeabilized or nonpermeabilized (as indicated above each graph) HEK293 cells transfected with the plasmids indicated in the insets of the right graph. These results are quantified in panel *D*. Bars represent the mean ± SD median fluorescence intensity (MFI) of three independent transfections from one experiment representative of three independent experiments. *p* values from a one-way ANOVA with Tukey correction for multiple comparisons are indicated.

Ccr1l1 conserves major structural and signaling features of bona fide chemokine GPCRs, and therefore, it is likely to bind chemokines and signal through G proteins if it can be expressed on the cell plasma membrane. To test this, we transiently transfected HEK293 cells with two different plasmid constructs, termed pNT1 and pNT2, containing the coding sequence of Ccr1l1 cloned from mouse splenic mRNA in frame with a C-terminal or N-terminal HA-tag, respectively (Fig. 4*A*). Protein expression was detected by fluorescence microscopy without membrane permeabilization (-Triton) after transfection with pNT2, whereas detection of the C-terminally tagged pNT1-encoded protein required permeabilization (+Triton, Fig. 4*B*). We confirmed and quantified these results by fluorescence-activated cell sorting (FACS) (Fig. 4, *C* and *D*). As shown in Figure 4*D*, comparable levels of pNT1-and pNT2-encoded proteins were detected in permeabilized cells, but only the N-terminally tagged pNT2-encoded protein was detectable in nonpermeabilized cells. Thus, Ccr1l1 can be expressed on the plasma membrane and contains an extracellular N terminus and an intracellular C terminus, consistent with a functional chemokine receptor.

### Ccr1l1 and Ccr1 differ in ligand-induced and constitutive signaling activities

**Figure 5:**
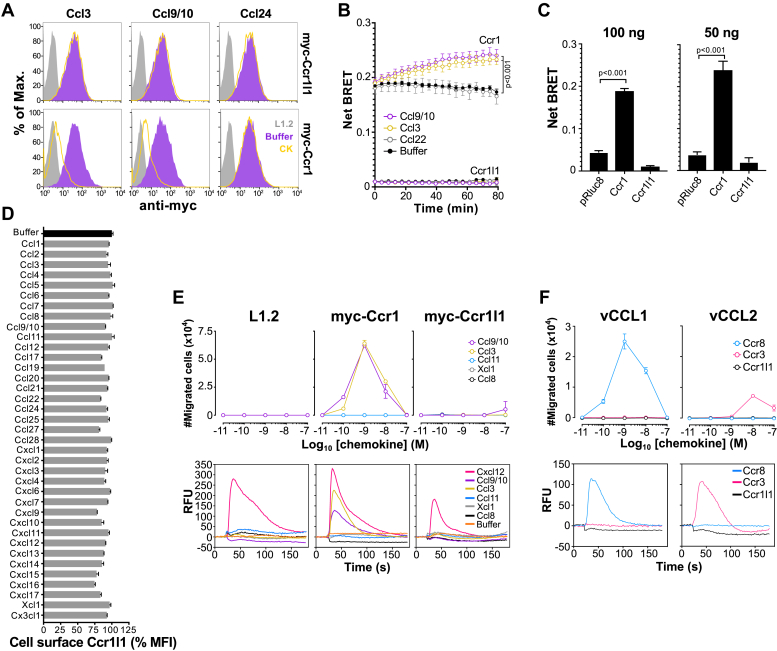
**Ccr1 but not Ccr1l1 is both constitutively active and chemokine-regulated.***A*, stable L1.2 cell lines expressing the receptors indicated on the right of each graph row were incubated with 100 nM of the chemokines indicated above each graph column. Levels of cell surface receptor were determined by FACS. Histograms for the staining with an anti-myc tag antibody of nonpermeabilized untransfected cells (L1.2, *gray*) or transfected cells after incubation with the corresponding chemokines (CK, *open yellow*) or buffer alone (*solid purple*) are shown. *B*, β-arrestin recruitment induced by buffer alone (*black*) or 100 nM of Ccl9/10 (*purple*), Ccl3 (*yellow*), or Ccl22 (*gray*) in Ccr1- or Ccr1l1-transfected 293T cells (as indicated near each group of curves) analyzed over time by BRET. *C*, Net BRET recorded over 20 min in 293T cells transfected with 50 ng or 100 ng (as indicated above each graph) of Ccr1- or Ccr1l1-expressing plasmids or pcDNA-Rluc8 (pRluc8, control plasmid) in the absence of chemokine. In both panels *B* and *C*, data are shown as mean ± SEM from triplicates of two independent experiments combined. *p* values from one-way ANOVA with Dunnet correction for multiple comparisons are indicated. *D*, cell surface Ccr1l1 after incubation of myc-Ccr1l1-expressing L1.2 cells with 100 nM of the indicated chemokines was detected by FACS as in panel *A*. Results were quantified as median fluorescence intensity (MFI) in % relative to cells incubated with buffer alone (*black bar*). Data are represented as the mean ± SD of duplicates from one experiment representative of three independent experiments. *E*, chemotaxis (top row) and calcium flux (bottom row) of untransfected (L1.2) and myc-Ccr1- and myc-Ccr1l1-expressing L1.2 cells (as indicated above each graph column) detected in response to the chemokines indicated in the insets of the right graphs. Cells were incubated with increasing doses of chemokine in chemotaxis assays or with 50 nM of chemokine or buffer alone injected at 20 s in calcium flux assays. Chemotaxis data are shown as the mean ± SD of the total number of migrated cells of duplicates from one experiment representative of three independent experiments. Results from calcium flux assays are shown as the relative fluorescence units (RFU) recorded over 180 s in one experiment representative of three independent experiments. *F*, chemotaxis (top row) and calcium flux (bottom row) induced by the viral chemokines vCCL1 and vCCL2 (as indicated above each graph column) in Ccr3-, Ccr8-, or myc-Ccr1l1-expressing L1.2 cells as indicated in the insets of the right graphs. Cells were incubated with chemokines as in panel *E*. Data are represented as in *E*. Chemotaxis data are from triplicates of one experiment representative of three independent experiments. Calcium flux data are from one experiment representative of three independent experiments.

In an attempt to uncover the ligand for Ccr1l1, we reasoned that it might act as an alternative receptor for Ccr1 ligands given its high level of amino acid identity with Ccr1. Moreover, closely related receptors for inflammatory chemokines all share some ligands, as do Ccr1 and Ccr3 (2). To test this hypothesis, we first analyzed the capacity of two well-established Ccr1 ligands, Ccl3 and Ccl9/10, to induce Ccr1l1 internalization and β-arrestin recruitment in Ccr1l1-expressing cells. For internalization experiments, we generated mouse L1.2 cell lines stably expressing myc-tagged Ccr1l1 (myc-Ccr1l1) and Ccr1 (myc-Ccr1). Of note, L1.2 cells have proved to be ideal for the study of chemokine-mediated responses and are a cellular background in which many orphan chemokine ligands and receptors have been deorphanized (45, 46, 47, 48, 49, 50). As shown in Figure 5*A*, both cell lines expressed high levels of the corresponding myc-tagged receptor on the plasma membrane and as expected, Ccl3 and Ccl9/10 but not Ccl24 (negative control) induced downregulation of cell surface Ccr1. However, none of these ligands affected surface expression of Ccr1l1 (Fig. 5*A*). To confirm this, we analyzed by bioluminescence resonance energy transfer (BRET) the β-arrestin recruitment induced by these chemokines in 293T cells transiently transfected with Ccr1l1 or Ccr1. We found that Ccl3 and Ccl9/10 but not Ccl22 (negative control) increased the net BRET signal over time in Ccr1-transfected cells (Fig. 5*B*). However, the net BRET signal after the addition of these chemokines did not diverge from that of the buffer in Ccr1l1-transfected cells (Fig. 5*B*). Interestingly, we observed that regardless of chemokine treatment, the baseline net BRET signal was higher in Ccr1-than in Ccr1l1-tranfected cells (Fig. 5*B*), suggesting constitutive β-arrestin recruitment by Ccr1. To address this more directly, we analyzed the net BRET signal in the absence of chemokine treatment after transfection with 100 or 50 ng of plasmids encoding Ccr1, Ccr1l1, or a control plasmid encoding only the Rluc8 reporter. While the net BRET recorded in Ccr1l1-transfected cells did not surpass that of cells transfected with the control plasmid, the signal was markedly higher in Ccr1-tranfected cells (Fig. 5*C*), indicating that mouse Ccr1 constitutively recruits β-arrestin in this system. It has been previously reported that human CCR1 is a constitutively active receptor (31, 51), but, to our knowledge, this is the first evidence of ligand-independent signaling by mouse Ccr1.

Since the Ccr1 ligands we tested induced neither internalization nor β-arrestin recruitment in Ccr1l1-expressing cells, we next tested all 37 commercially available mouse chemokines on the myc-Ccr1l1 cell line using receptor internalization, calcium flux, and chemotaxis assays. As shown in Figure 5, *D* and *E*, none of the mouse chemokines tested was able to induce a functional response in any of the three assays for Ccr1l1. None of the 37 mouse chemokines downregulated the levels of cell surface Ccr1l1 in the myc-Ccr1l1 cell line (Fig. 5*D*) or induced a detectable migration or calcium flux response in these cells (Fig. 5*E*). As expected, Ccl3 and Ccl9/10 induced chemotaxis and calcium mobilization in myc-Ccr1-expressing cells (Fig. 5*E*). Importantly, Cxcl12 (ligand for Cxcr4, a receptor endogenously expressed by L1.2 cells) induced calcium mobilization in untransfected as well as myc-Ccr1-and myc-Ccr1l1-expressing L1.2 cells (Fig. 5*E*, bottom), confirming that all three cell lines were capable of responding in this type of assay. Of note, all chemokines used in the internalization assays of Figure 5*D* were also tested in chemotaxis and calcium flux assays, but only selected chemokines are presented in Figure 5*E* for clarity purposes. Therefore, we concluded that there is no conventional chemokine ligand for Ccr1l1 among the 37 known and commercially available mouse chemokines.

Many viruses, especially in the *Herpesviridae* family, encode chemokine homologs to hijack the chemokine antiviral response of their hosts (33, 52). vCCL1 and vCCL2 (also termed vMIP-I and vMIP-II, respectively) expressed by the Kaposi's sarcoma-associated herpesvirus are among the best characterized viral chemokines. vCCL1 is a specific agonist for human CCR8, whereas vCCL2 is a broad range chemokine receptor antagonist that has also been reported to selectively agonize human CCR3 and CCR8 (53, 54, 55, 56). We decided to test whether these two viral chemokines were able to activate Ccr1l1 in chemotaxis and calcium flux assays. To validate the activity of recombinant vCCL1 and vCCL2, we included as controls two stable L1.2 lines existing in our laboratory for mouse Ccr3 and Ccr8. As shown in Figure 5*F*, and consistent with its reported activity, vCCL1 induced migration and calcium mobilization in Ccr8-expressing cells, but not in Ccr3-or Ccr1l1-expressing cells. On the other hand, vCCL2 induced a strong calcium signal in Ccr3-expressing cells, but not in Ccr1l1- or Ccr8-expressing cells (Fig. 5*F*, bottom). Also, although the efficacy was low, only Ccr3-expressing cells showed detectable and reproducible migration in response to vCCL2, with the migration peak at 10 nM of chemokine (Fig. 5*F*, top). The inability of vCCL2 to activate mouse Ccr8 in our experiments may indicate that this viral chemokine is specific for the human counterpart of this receptor. However, it is important to note that, unlike its CCR3-mediated activity, which has been independently confirmed by several groups and now also by us with mouse Ccr3, the vCCL2 action at CCR8 remains unclear (57, 58). We concluded that Ccr1l1 is not activated by vCCL1 or vCCL2. It will be interesting to investigate whether other viral chemokines, especially those expressed by rodent-specific viruses, are able to signal through Ccr1l1.

### Incorporation of a sulfatable N-terminal tyrosine essential for Ccr1 signaling does not confer chemokine interaction to Ccr1l1

**Figure 6:**
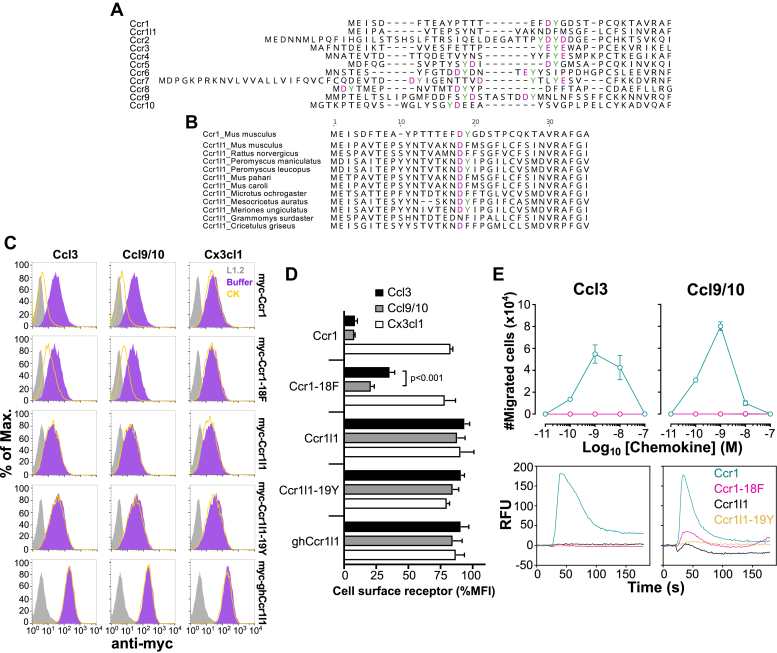
**A sulfatable tyrosine in the N terminus of Ccr1 modulates ligand recognition and signaling, but absence of this residue does not explain lack of recognition of these ligands by Ccr1l1.***A*, conservation of sulfatable tyrosines in the N-terminal domain of mouse CC chemokine receptors but not mouse Ccr1l1. Sequences of the N-terminal domains of the indicated mouse receptors were aligned using MUSCLE. Tyrosines (highlighted in *green*) are more likely to be sulfated when flanked by acidic residues (D or E, highlighted in *magenta*). *B*, partial conservation of a sulfatable tyrosine in the N-terminal domain of rodent Ccr1l1. Sequences from the indicated Ccr1l1 rodent species were aligned by MUSCLE. The only tyrosine (Y^18^) flanked by an acidic residue (D^17^, highlighted in *magenta*) in Ccr1 is highlighted in *green*. *C*, Ccr1l1 variants containing an N-terminal sulfatable tyrosine do not internalize in response to Ccr1 ligands. Stable L1.2 cells expressing myc-tagged mouse Ccr1, the mutant Ccr1 Y18F (myc-Ccr1-18F), mouse Ccr1l1, the variant Ccr1l1 F19Y (myc-Ccr1l1-19Y), or golden hamster Ccr1l1 (myc-ghCcr1l1), which naturally contains a sulfatable tyrosine in its N terminus (see “Ccr1l1_*Mesocricetus auratus*” in panel *B*), were incubated with 100 nM of the chemokines indicated above each graph column. Then, levels of cell surface receptor were analyzed by FACS. Histograms for the staining with an anti-myc tag antibody of nonpermeabilized untransfected cells (L1.2, *gray*) or transfected cells after incubation with the corresponding chemokines (CK, *open yellow*) or buffer alone (*solid purple*) are shown. *D*, Ccr1 Y^18^ differentially contributes to Ccl3 and Ccl9/10 interaction. Quantification of cell surface receptor levels after incubation of the L1.2 cell lines used in panel *C* with the chemokines indicated in the legend. Data are shown as the mean ± SD of the median fluorescence intensity (MFI) in % relative to cells incubated with buffer alone. Data are from triplicates of two independent experiments whose results were combined. The indicated *p* value is from a two-way ANOVA analysis with Tukey correction for multiple comparisons. *E*, sulfatable Y^18^ is essential for Ccr1 signaling but does not suffice to equip Ccr1l1 with Ccr1-like activity. Chemotaxis (top) and calcium flux assays (bottom) with L1.2 cell lines expressing the receptors indicated in the *inset* of the bottom right graph in response to Ccl3 and Ccl9/10 (as indicated above each graph column). In chemotaxis assays cells were incubated in the presence of increasing doses of chemokines and data are represented as the mean ± SD total migrated cells of triplicates from one experiment representative of three independent experiments. In calcium flux assays 50 nM of chemokine was injected at 20 s and the relative fluorescence units (RFU) were monitored over 180 s. Data are from one experiment representative of three independent experiments.

To understand the structural basis for why Ccr1 ligands fail to interact with Ccr1l1 despite >70% overall amino acid identity for the receptors, we focused on differences in the N-terminal domain, which, structural studies have shown, forms an extended ligand-binding interface for chemokine receptors (16, 17, 19). In particular, N-terminal sulfotyrosine residues play a prominent role in the regulation of the ligand-binding affinity and selectivity of chemokine receptors and other GPCRs (22, 59, 60, 61). Acidic residue/tyrosine motifs are known to be preferred sites for tyrosine sulfation (21). All mouse CC chemokine receptors contain at least one tyrosine flanked by aspartic acid (D) or glutamic acid (E) in their N-terminal domain (Fig. 6*A*). In stark contrast, we noticed that the N-terminal domain of mouse Ccr1l1 does not present any tyrosine adjacent to acidic residues (Fig. 6*A*). In fact, a Ccr1 DY motif (D^17^ and Y^18^ of Ccr1) is replaced most commonly with DF in seven of 11 Ccr1l1 rodent species, including mouse Ccr1l1, whereas it is conserved in the other four species (Fig. 6*B*). The phenylalanine replacement in mouse Ccr1l1 would preserve most physical properties of a tyrosine, but it cannot be sulfated since it lacks the necessary hydroxyl group. To test the significance of this anomaly in mouse Ccr1l1, we generated stable L1.2 cell lines expressing an F19Y variant of mouse Ccr1l1 (myc-Ccr1l1-19Y) and golden hamster (*Mesocricetus auratus*) Ccr1l1 (myc-ghCcr1l1), one of the four rodent Ccrl1l species that conserves the N-terminal DY motif (Fig. 6*B*). Also, both as a control and to investigate the role of Y^18^ in ligand binding by Ccr1, we generated a stable cell line for a Y18F Ccr1 mutant (myc-Ccr1-18F). As shown in Figure 6*C*, all cell lines expressed high levels of the corresponding receptors on the cell surface. Consistent with the experiments in Figure 5, Ccl3 and Ccl9/10 but not a control chemokine (Cx3cl1) induced efficient internalization of wild-type myc-Ccr1 (Fig. 6*C*). In contrast, internalization of myc-Ccr1-18F was reduced compared with myc-Ccr1 in response to both Ccr1 ligands, pointing to the functional importance of Ccr1 Y^18^ in ligand interaction, possibly due to tyrosine sulfation of the site (Fig. 6, *C* and *D*). Importantly, myc-Ccr1-18F was more resistant to Ccl3-mediated internalization (35% cell surface receptor) than to Ccl9/10-mediated internalization (20% cell surface receptor) (Fig. 6*D*), suggesting that Y^18^ plays a more decisive role in the Ccr1-Ccl3 complex than in the Ccr1-Ccl9/10 complex. However, F19Y substitution in mouse Ccr1l1 failed to confer any internalization of the receptor in response to Ccr1 ligands (Fig. 6, *C* and *D*). Similarly, cell surface ghCcr1l1, despite its conserved N-terminal DY motif, was not downregulated by Ccl3 or Ccl9/10 (Fig. 6, *C* and *D*). Therefore, we concluded that the absence of a sulfatable tyrosine in the N terminus of Ccr1l1 does not fully explain its inability to interact with Ccr1 ligands.

To evaluate the role of Ccr1 Y^18^ in receptor signaling, we performed chemotaxis and calcium flux assays using these N-terminal tyrosine mutant receptor-expressing cell lines. As expected, Ccl3 and Ccl9/10 induced strong chemotactic and calcium responses in Ccr1-expressing cells, whereas, consistent with the internalization data of Figure 6*D*, no response was detected in Ccr1l1-or Ccr1l1-19Y-expressing cells (Fig. 6*E*). However, with the only exception of a small calcium flux bump induced by Ccl9/10 (Fig. 6*E*, bottom), the Y18F substitution nearly abolished Ccr1 chemotactic and calcium responses (Fig. 6*E*). Therefore, although Ccr1-18F was still capable of chemokine binding to some extent, as indicated by the internalization experiments (Fig. 6*D*), we concluded that Y^18^ is essential for Ccr1 signaling.

### Ccr1l1 deficiency does not affect the activity of Ccr1, Ccr3, or other chemokine receptors in primary bone marrow-derived eosinophils

**Figure 7:**
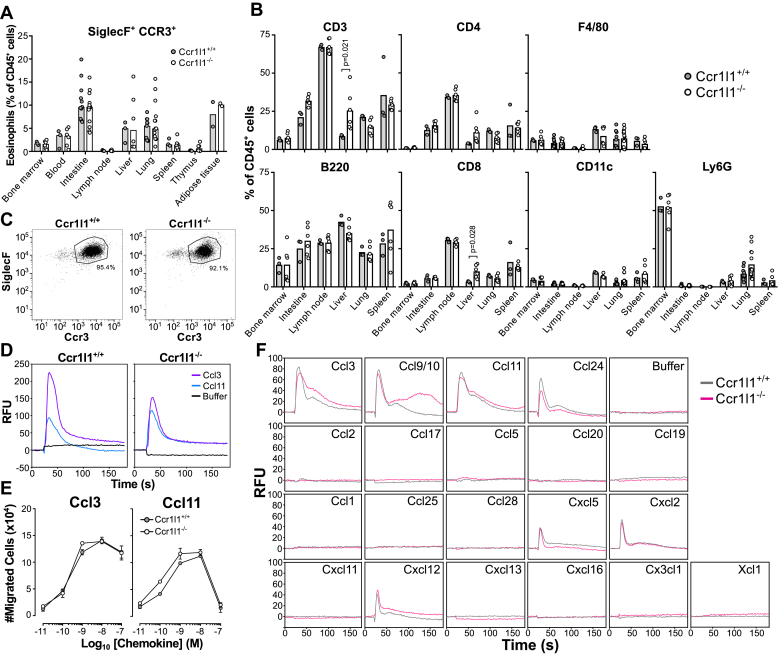
**Ccr1l1 deficiency does not alter mouse immune cell populations or chemokine-induced responses in mouse bone-marrow-derived eosinophils (BMDE)**. *A*, *Ccr1l1*^*−/−*^ eosinophil phenotyping. Eosinophils were analyzed by FACS in various tissues (*x*-axis) from naïve *Ccr1l1*^*−/−*^ and *Ccr1l1*^*+/+*^ mice (n = 2–14) as indicated in the *inset*. Results are represented as the % of eosinophils among CD45^+^ leukocytes. *Dots* represent individual mice and bars indicate median % values. *B*, immunophenotyping of *Ccr1l1*^*−/−*^ mice. The immune cell populations indicated above each graph were analyzed by FACS in various tissues (*x*-axis) from naïve *Ccr1l1*^*−/−*^ and *Ccr1l1*^*+/+*^ (n = 3–14) as indicated in the legend. Results are represented as % of CD45^+^ leukocytes. Dots represent individual mice and bars indicate mean %. *p* values for statistically significant (*p* < 0.05) differences detected between *Ccr1l1*^*−/−*^ and *Ccr1l1*^*+/+*^ mice by multiple *t* tests with Holm-Sidak correction for multiple comparisons are indicated. *C*, differentiation of BMDE *in vitro* is not affected by the absence of Ccr1l1. BMDE were generated *in vitro* by culture of bone marrow cells from *Ccr1l1*^*−/−*^ or *Ccr1l1*^*+/+*^ mice (as indicated above each graph) in the presence of IL-5. Dot plots show the purity of final BMDE samples measured as the % of SiglecF+ Ccr3+ cells. The ability of *Ccr1l1*^*−/−*^ and *Ccr1l1*^*+/+*^ BMDE to respond to the Ccr1 ligand, Ccl3, and the Ccr3 ligand, Ccl11, was analyzed by calcium flux (*D*) and chemotaxis assays (*E*). In *D*, calcium mobilization after addition (at 20 s) of buffer alone (*black*), or 50 nM of Ccl3 (*purple*) or Ccl11 (*blue*) was monitored as the relative fluorescence units (RFU) for 180 s in *Ccr1l1*^*−/−*^ and *Ccr1l1*^*+/+*^ BMDE (as indicated above each graph). Data are from one experiment representative of three independent experiments. In *E*, transwell chemotaxis assays of *Ccr1l1*^*−/−*^ and *Ccr1l1*^*+/+*^ BMDE (as indicated in the *inset*) in response to increasing concentrations (*x*-axis) of Ccl3 or Ccl11 (as indicated above each graph). Data are presented as the mean ± SD of the total number of migrated cells of duplicates from one experiment representative of three independent experiments. *F*, screening of chemokine-induced calcium flux in *Ccr1l1*^*−/−*^ BMDE. Calcium mobilization after addition of 50 nM of the indicated chemokines or buffer alone was recorded in *Ccr1l1*^*−/−*^ and *Ccr1l1*^*+/+*^ BMDE (as indicated in the legend) as in panel *D*. Data are from one experiment representative of three independent experiments.

Increasing evidence indicates that chemokine receptors can also regulate chemokine-mediated bioactivities by the formation of heterodimers with other chemokine receptors (36). We have shown that Ccr1l1 is primarily expressed by mouse eosinophils (Fig. 2), which are known to express mainly Ccr1 and Ccr3 (62); intriguingly, the two chemokine receptors most highly related to Ccr1l1. Therefore, we next tested whether the absence of Ccr1l1 altered the signaling mediated by Ccr1 and Ccr3. For this, using BMDE, which, we have shown, express high levels of Ccr1l1 (Fig. 2*D*), we compared the chemotactic and calcium flux responses to Ccl3 (Ccr1 ligand) and Ccl11 (Ccr3 ligand) in BMDE obtained from *Ccr1l1*^*+/+*^ and *Ccr1l1*^*−/−*^ mice. Importantly, naïve *Ccr1l1*^*−/−*^ mice displayed normal tissue eosinophil phenotypes (Fig. 7*A*) and overall unaltered immune cell populations, with the exception of slightly elevated T cells in the liver (Fig. 7*B*). Furthermore, absence of Ccr1l1 did not affect the development of BMDE, identified as Ccr3^+^ SiglecF^+^ cells (Fig. 7*C*), from bone marrow cells stimulated with IL-5. Both *Ccr1l1*^*+/+*^ and *Ccr1l1*^*−/−*^ BMDE gave strong calcium responses after stimulation with Ccl3 and Ccl11 (Fig. 7*D*). In chemotaxis experiments, no differences in the efficacy and potency of Ccl3 and Ccl11 to attract *Ccr1l1*^*+/+*^ or *Ccr1l1*^*−/−*^ BMDE were observed (Fig. 7*E*). To explore the possibility that Ccr1l1 may interfere with other eosinophil chemokine receptors, we extended the calcium flux analysis of BMDE to 20 different chemokines, including at least one ligand for every known mouse chemokine receptor and the Ccr1/Ccr3 ligands Ccl3, Ccl9/10, Ccl11, and Ccl24 as controls. Both *Ccr1l1*^*+/+*^ and *Ccr1l1*^*−/−*^ BMDE displayed the same chemokine response profile (Fig. 7*F*). In addition to the Ccr1 and Ccr3 agonists, Cxcl12 (ligand for the ubiquitous Cxcr4 receptor), Cxcl5, and Cxcl2 induced comparable calcium flux responses in both types of BMDE (Fig. 7*F*). Cxcl2 is a specific ligand for Cxcr2, whereas Cxcl5 activates Cxcr1 and Cxcr2; both receptors have been reported to be expressed in IL-5 primed eosinophils (63). Therefore, we concluded that Ccr1l1 may not functionally interact with other chemokine receptors in mouse eosinophils.

## Discussion

Ccr1l1 has remained an orphan member of the chemokine receptor family since it was cloned in 1995 (39). In this study, we demonstrate for the first time that mouse Ccr1l1 is an integral plasma membrane protein selectively expressed in eosinophils that preserves all major structural components of signaling chemokine receptors, except for an N-terminal sulfatable tyrosine. We ruled out the viral chemokines vCCL1 and vCCL2 and all currently known mouse chemokines as agonists of Ccr1l1 in multiple classic chemokine GPCR signaling assays. Furthermore, we showed that Ccr1l1 does not exert ligand-independent activities such as constitutive β-arrestin recruitment or trans-regulation of Ccr1 and Ccr3. In addition, although it was included in our study as a control receptor for its close relation to Ccr1l1, here we unveil novel aspects of the activity and ligand-binding determinants of mouse Ccr1. In particular, we demonstrate that mouse Ccr1 is a constitutively active receptor and that its conserved N-terminal tyrosine is differentially involved in receptor binding to Ccl3 and Ccl9/10 but it is equally important for the cellular responses induced by these ligands. In contrast, this key N-terminal tyrosine is absent in Ccr1l1, and its incorporation by mutagenesis does not confer Ccr1-like activity to Ccr1l1. Further investigation will be required to identify the putative bioactivity of Ccr1l1.

This negative screen was unexpected given that Ccr1l1 shares 70% identity with Ccr1 and 50% identity with Ccr3, both of which have highly promiscuous ligand relationships, several of which are shared between the two receptors. Nevertheless, it is important to note that primary sequence relationships may be misleading when it comes to predicting chemokine ligand specificities and signaling pathways for even highly promiscuous receptors. For instance, ACKR1, which shares only 25% amino acid identity at most with other chemokine receptors, has the broadest ligand repertoire of any human chemokine receptor family member, binding to many but not all CC and CXC chemokines (30). Also, viral chemokine receptors are able to bind with high affinity many human chemokines despite being highly divergent from human chemokine receptors (33). Conversely, although mouse Cxcr1 shares about 70% amino acid identity with its human counterpart, it took nearly 20 years after human CXCR1 was first cloned to identify the first bona fide ligand for mouse Cxcr1 (64, 65, 66). Moreover, human CXCR1 and CXCR2 are 79% identical at the amino acid level, yet CXCR2 has seven known high-affinity chemokine ligands, only two of which are shared with CXCR1 (CXCL6 and CXCL8) (2, 67). Furthermore, there is no mouse counterpart of CXCL8, and mouse Cxcr1 has no known high-affinity chemokine ligand, just two low-affinity ligands (64).

Regardless of the primary amino acid sequence, key structural chemokine-binding determinants are well conserved in all functional chemokine receptors. For instance, the ECL2 β-hairpin has been reported to be directly involved in ligand binding by CCR6, CXCR4, ACKR3, and the viral GPCR US28 (16, 17, 68, 69, 70). In addition, two extracellular disulfide bridges, one between TMVII and the N terminus and the other between TMII and ECL2, have been shown to be essential for chemokine binding and activation of many chemokine receptors (6, 7, 8, 9, 10, 11). Here, we show that these critical functional determinants of the tertiary structure of chemokine receptors as well as the core topology (extracellular N terminus and intracellular C terminus) of this receptor family are conserved in Ccr1l1. Furthermore, highly conserved amino acid residues and motifs involved in signaling initiation, signal transmission, and receptor activation in GPCRs, including the signature G-protein-coupling DRY motif, are also preserved in the sequence of Ccr1l1. Since ACKRs typically lack one (mainly the DRY motif) or more of these signaling motifs, these findings support that Ccr1l1 is more likely to act as a signaling GPCR than as a β-arrestin-biased ACKR. However, future studies will be necessary to substantiate this *via* a comprehensive β-arrestin screening of Ccr1l1, extending the one we have performed for the present study. In particular, evidence that this GPCR homolog binds chemokines will be required before it can be definitively designated as a member of either the chemokine GPCR or ACKR families.

The only obvious glitch we found in the sequence of Ccr1l1 was the absence of a tyrosine sulfation motif in its N terminus. N-terminal sulfotyrosines are known to enhance chemokine-binding affinity and regulate ligand selectivity for chemokine receptors (22). Consistent with this, we demonstrate here that a Ccr1 Y18F mutant displays reduced internalization capacity in response to Ccl3 and Ccl9/10, with Ccl3-mediated internalization being more significantly affected by this mutation. Of note, downregulation of cell surface Ccr1 Y18F upon incubation with Ccl3 and Ccl9/10 was hindered but not abolished, which denotes that this Ccr1 variant preserves some ligand-binding capabilities. In spite of this, we found that Ccr1 Y18F was unable to initiate chemotactic or calcium responses, indicating that Y^18^ is important for Ccr1 signaling.

We are just beginning to understand the role of the N-terminal domain and in particular N-terminal sulfotyrosines in the signaling activity of chemokine receptors. Unlike the classical two-step/two-site model that reserves the signaling regulation for the transmembrane bundle of the receptor, several recent reports have shown that the N-terminal domain does not solely control the ligand-binding affinity and selectivity, but it is also significantly implicated in the modulation of receptor signaling (71, 72, 73). Our results support the signaling functions of the N terminus and assign a decisive role in the ligand binding and signaling activity of mouse Ccr1 to its N-terminal sulfotyrosine. Similarly, it has been reported that a CXCR3 mutant with its N-terminal tyrosines swapped to phenylalanine does not migrate in response to CXCL11 stimulation despite preserving 50% of the internalization activity of wild-type CXCR3 (74). In contrast, similar mutations in CXCR4 or CXCR6 had no effect on agonist-induced receptor–G protein association or chemotaxis, respectively (72, 75). Hence, the signaling implications of N-terminal sulfotyrosines appear to vary greatly among chemokine receptors; therefore, their absence in the N terminus of Ccr1l1 does not necessarily render this receptor inactive. In fact, human CXCR2 and CXCR5, receptors with well-established ligands, do not contain a clear sulfation motif in their N-termini either (21). It will be important to establish the role of N-terminal sulfotyrosines in the signaling activity of other chemokine receptors. Probably biased by the traditional role assigned to the N-terminal domain, most previous studies were limited to understanding how N-terminal sulfotyrosines regulate the chemokine-binding properties of chemokine receptors. Our work highlights the importance of extending these analyses to functional assays such as chemotaxis or calcium mobilization.

Our data suggest that Ccr1l1 is not a pseudogene, but instead encodes a viable and stable protein able to traffic to the plasma membrane. Although it is possible that Ccr1l1 is in transition to becoming a pseudogene, this seems unlikely considering that it appears to have evolved late in mammalian phylogeny and directly from Ccr1, that it is remarkably conserved in rodents but is not found in any other animal order, and that it is selectively expressed in eosinophils. Instead, these observations suggest that Ccr1l1 may play significant roles in the biology of rodent eosinophils. Nevertheless, we found that naïve *Ccr1l1*^*−/−*^ mice have normal eosinophil frequencies in tissues and that the absence of Ccr1l1 does not affect Ccr1-, Ccr3-, Cxcr4, and Cxcr2-mediated signaling in BMDE. Still, as previously reported for other chemokine receptors, the immunological phenotype of *Ccr1l1*^*−/−*^ mice might only become apparent during infection or inflammation. In this regard, future experiments using *Ccr1l1*^*−/−*^ mice focusing on eosinophilic models, such as parasitic and allergic challenges, may help to characterize the immune functions of Ccr1l1. In addition, as a strategy to identify Ccr1l1 ligands, it will be interesting to test whether extracts from eosinophilic tissues activate Ccr1l1-expressing cells. Of note, these future directions would benefit significantly from the development of an anti-Ccr1l1 antibody that is currently unavailable. Since research relies greatly on mouse models to understand the role of chemokines in immunity and inflammation and to identify new chemokine-based clinical applications, it will be important to decipher how Ccr1l1, the only putative member of the known mouse chemokine receptor family without a human ortholog, may differentially shape the mouse immune response from that of humans, especially where eosinophils are involved.

## Experimental procedures

### Animals

C57BL/6NTac mice were obtained from Taconic Biosciences. All mice were maintained under specific pathogen-free housing conditions at an American Association for the Accreditation of Laboratory Animal Care—accredited animal facility at the National Institute of Allergy and Infectious Diseases (NIAID) and housed in accordance with the procedures outlined in the Guide for the Care and Use of Laboratory Animals under the protocol LMI-8E approved on 12/31/2015 and annually renewed by the Animal Care and Use Committee of NIAID.

### Cells and reagents

L1.2 cells (kindly provided by Dr Eugene Butcher, Stanford University) were grown in RPMI-GlutaMAX medium supplemented with 10% FBS, 0.1 mM nonessential amino acids, 1 mM sodium pyruvate, 55 μM 2-mercaptoethanol, and 100 U/ml penicillin-streptomycin (all from Life Technologies). HEK293 and 293T cells (ATCC) were grown in DMEM-GlutaMax medium (Life Technologies) supplemented with 10% FBS. All cells were maintained at 37 °C, 5% CO_2_, and 100% humidity. Recombinant mouse chemokines were from R&D Systems or PeproTech and reconstituted and stored per the manufacturer's recommendations. Recombinant viral chemokines vCCL1 and vCCL2 were purchased from R&D Systems.

### Ccr1l1 knockout mice

Ccr1l1-deficient mice were generated from Taconic C57BL/6NTac mice using the CRISPR-Cas9 genome-editing method and the small RNA guides Ccr1l1-sgRNA1 (gctttgctattaaacacgtaggg) and Ccr1l1-sgRNA3 (ggtatctatcaatcgtaagcagg). Five of 31 pups were confirmed to be homozygous for the mutation by PCR using the primers molMR4316 (gacccaggtaatgatgctactgatg), molMR4317 (cagggtttagtaaactctaggc), and molMR4318 (gacaatgacctttatatttgc). By DNA sequencing, we found that four Ccr1l1 knockout founder mice had the same deletion from position –58 to +387 relative to the ATG initiation codon, and one mouse had a deletion between –61 and +428. Therefore, in these five mice, our CRISPR-Cas9 editing of Ccr1l1 resulted in the elimination of the initiation codon and at least the first 129 residues of the protein. All five mice were bred as separate *Ccr1l1*^*−/−*^ lines, and all experiments were reproduced with at least two independent knockout lines.

### Immunophenotyping of Ccr1l1 knockout mice

Eosinophil and other immune cell populations in different tissues from *Ccr1l1*^*−/−*^ and *Ccr1l1*^*+/+*^ mice were analyzed by FACS. Tissues of interest were harvested and placed in phosphate-buffered saline (PBS) on ice. Blood was collected by cardiac puncture and red blood cells were immediately lysed using ammonium-chloride-potassium (ACK) lysing buffer. Single-cell suspensions from spleen, lymph nodes, and thymus were generated by pressing the tissues through 70 μm cell strainers. Bone marrow cells were collected from femurs and tibias by flushing with 5 ml of cold PBS. Lungs and liver were collected, minced, resuspended in digestion media (FBS-free DMEM-GlutaMax media containing 0.1 mg/ml Liberase TL and 20 μg/ml DNAseI [enzymes from Sigma]), and incubated for 1 h at 37 °C. Cells from the small intestine were isolated essentially as previously described from the colon (76). Briefly, small intestines were collected while removing as much fat as possible and placed into buffer A (HBSS, 5% FCS, 25 mM HEPES). Intestines were cleaned out, opened up, shaken in buffer A, and strained. Mucus was removed after cutting the tissue into 2 cm segments, shaking in buffer A, and straining. Segments were transferred to cold buffer B (HBSS, 2 mM EDTA, 25 mM HEPES), vortexed, and strained. The tissue was incubated for 15 min at 37 °C with agitation in 10 ml prewarmed buffer C (HBSS, 15 mM HEPES, 5 mM EDTA, 10% FCS, 0.015% DTT), with vortex every 5 min and at the end of incubation. The epithelial layer was then removed by shaking and straining five times in buffer A. Samples were centrifuged and digested in media (IMDM [Life Technologies] supplemented with 0.17 mg/ml Liberase TL, DNaseI 30 μg/ml, 10% FBS, 100 U/ml penicillin-streptomycin, 100 U/ml glutamine, 15 mM HEPES) for 60 min at 37 °C on a shaker at 200 rpm. Samples were centrifuged, resuspended in cold buffer A for 2 min, and centrifuged again. Finally, tissue was pressed through a 100 μm cell strainer, centrifuged, resuspended in buffer B, and filtered through a 40 μm cell strainer. Cells from both posterior subcutaneous adipose tissue depots were isolated as previously described (77). Briefly, the tissue was minced, resuspended in 5 ml of digestion media (DMEM/F12-GlutaMax media [Life Technologies] supplemented with 2% FBS, 0.1 mg/ml Liberase TM, and 0.1 mg/ml DNaseI [enzymes from Sigma]), and incubated with agitation at 200 rpm for 40 min at 37 °C.

Single-cell suspensions were centrifuged, red blood cells were lysed using ACK buffer, and cells were resuspended in PBS-staining buffer (1X PBS supplemented with 1% FBS and 1% BSA). Approximately one million cells/sample were stained for FACS analysis. Before antibody staining, cellular Fc receptors were blocked using TruStain FcX (Biolegend). Cells were stained in PBS-staining buffer with APC-Cy7-conjugated rat anti-mouse CD45 (clone 30F11, Biolegend), PerCP-eFluor710-conjugated rat anti-mouse SiglecF (clone 1RNM44N, eBioscience), BUV395-conjugated rat anti-mouse CD11b (clone M1/70, BD Bioscience), PE-conjugated rat anti-mouse Ccr3 (clone J073E5, Biolegend), APC-conjugated rat anti-mouse F4/80 (clone BM8, eBioscience), BV785-conjugated rat anti-mouse Ly6G (clone 1A8, Biolegend), FITC-conjugated hamster anti-mouse CD11c (clone N418, eBioscience), BV650-conjugated hamster anti-mouse CD3ε (clone 145-2C11, BD Bioscience), FITC-conjugated rat anti-mouse CD4 (clone RM4-5, eBioscience), PE-conjugated rat anti-mouse CD8α (clone 53–6.7, Biolegend), BV785-conjugated rat anti-mouse B220 (clone RA3-6B2, Biolegend), and Fixable Viability Dye eFluor450 (eBioscience). In total, 100,000 events were collected on a BD Fortessa cytometer, and data were analyzed using FlowJo (both from BD Biosciences). Cell duplets and aggregates were excluded by SSC-H *versus* SSC-A gating and only live cells, negative for the viability staining, were included in the analysis. Eosinophils were quantified as the % of CD11b^+^ CCR3^+^ SiglecF^+^ cells in a gate of viable CD45^+^ cells. Other populations of interest included B cells (“B220”, identified as B220^+^ CD3^-^ cells), T cells (“CD3”, B220^-^ CD3ε^+^), CD4 cells (“CD4”, B220^-^ CD3ε^+^ CD8α^-^ CD4^+^), CD8 cells (“CD8”, B220^-^ CD3ε^+^ CD8α^+^ CD4^-^), macrophages (“F4/80”, CD11b^+^ F4/80^+^), dendritic cells (“CD11c”, CD11b^+/-^ CD11c^+^), and neutrophils (“Ly6G”, CD11b^+^ Ly6G^+^ SiglecF^-^).

### Generation of bone marrow-derived macrophages (BMDM) and eosinophils (BMDE)

BMDM and BMDE were differentiated from bone marrow cells of *Ccr1l1*^*+/+*^ or *Ccr1l1*^*−/−*^ mice. Bone marrow was harvested from tibias and femurs by flushing and straining the opened bones with PBS. For the generation of BMDM, five million bone marrow cells were cultured in a 100 mm petri dish in RPMI-GlutaMax media supplemented with 20% FBS and 30% L929-conditioned media. Fresh media was added 3 days after isolation and cells were cultured for four additional days before experiments. For the generation of BMDE, after lysis of red blood cells with ACK buffer, the leukocyte fraction was separated by centrifugation (20 min, 1200 x *g*) of bone marrow cells in Lympholyte-M separation media (Cedarlane). Cells at the interface were collected, counted, and cultured at 10^7^ cells/ml in BMDE media (RPMI-GlutaMax, 20% FBS, 1x nonessential amino acids, 100 U/ml penicillin-streptomycin, 1 mM sodium pyruvate, 55 μM 2-mecaptoethanol) supplemented with 100 ng/ml stem cell factor and 100 ng/ml Flt-3 ligand (both from Peprotech). Two days later, an equal volume of fresh BMDE media supplemented with stem cell factor and Flt-3 ligand was layered on top of the cells. On day 4, cells were moved to new flasks at 3 to 5 × 10^6^ cells/ml in BMDE media supplemented with 10 ng/ml of recombinant mouse IL-5 (mIL-5, Peprotech). On day 7, the cells were moved again to new flasks at 1 × 10^6^/ml in BMDE media containing mIL-5. On days 9 and 11, half of the media was replaced with fresh BMDE media supplemented with mIL-5. On day 14, cells were collected for experiments. To confirm the purity of our BMDE cultures, 10^6^ cells were washed with PBS-staining buffer (1X PBS, 1% FBS, and 1% BSA) and stained with PerCP-eFluor710-conjugated rat anti-mouse SiglecF (clone IRNM44N, Life Technologies) and PE-conjugated rat anti-mouse Ccr3 (clone J073E5, Biolegend). Data were collected on a BD Fortessa cytometer and analyzed using FlowJo (both from BD Biosciences).

### Ccr1l1 gene expression

Mouse tissues were homogenized in 1 ml of PBS using a tissue homogenizer (Omni International), and 100 μl of the homogenate was rapidly mixed with 1 ml of Trizol (Life Technologies). Splenocytes were sorted into B, T, and non-T/non-B cells using mouse CD19 and CD90.2 microbeads in an autoMACS Pro separator (all from Miltenyi Biotech). Cell pellets of sorted splenocyte populations as well as those of BMDE and BMDM were directly lysed with 1 ml Trizol. After chloroform extraction, the RNA-containing clear phase was diluted with an equal volume of 70% ethanol, and total RNA was purified using the Isolate II RNA mini kit (Bioline). Contaminant genomic DNA was eliminated from RNA samples using the Turbo DNA-free kit (Life Technologies), and cDNA was obtained from 100 to 500 ng of RNA using the SensiFast cDNA synthesis kit (Bioline). Where indicated, separate RNA samples were mock-treated without reverse transcriptase. The expression of Ccr1l1 was calculated relative to the expression of Gapdh by qPCR. The Cq values for gene amplification were obtained in a CFX96 Real-Time System (Bio-Rad) using the SensiFast SYBR kit (Bioline) and the following primer pairs: Gapdh F (aactttggcattgtggaagg) and Gapdh R (acacattgggggtaggaaca); Ccr1l1qPCR F2 (acacacacttcagagacgtga) and Ccr1l1qPCR R2 (tgtgacagctggaatctccatt). Ccr1l1 relative expression was calculated as 2^(Cq[Ccr1l1]-Cq[Gapdh])^.

### Cloning and mutagenesis of Ccr1- and Ccr1l1-expressing plasmids

The complete ORF of mouse Ccr1l1 was obtained by RT-PCR from mRNA isolated from splenocytes of C57BL/6NTac mice (Taconic). For transient expression in HEK293 cells, Ccr1l1 was amplified from splenic cDNA by PCR to incorporate a Kozak sequence and a C-terminal or N-terminal HA tag using the primer pairs Ccr1l1 F1/R1 (ccgcttaagccaccatggagattccagctgtc/ccgtctagattaagcataatctggaacatcatatggatataagtcggcagaggtctc) or Ccr1l1 F2/R2 (gcccttaagccaccatggcgtatccatatgatgttccagattatgctgagattccagctgtcacag/ccgtctagattataagtcggcagaggtctc), respectively. PCR fragments were cloned into a pcDNA3.1 plasmid resulting in the plasmids pNT1 (HA tag at the C terminus) and pNT2 (HA tag at the N terminus). For the generation of stable L1.2 cell lines, mouse Ccr1l1, golden hamster Ccr1l1 (ghCcr1l1), and mouse Ccr1 were cloned in frame with a myc tag at the N terminus. For this, Ccr1l1 was amplified by PCR from pNT2 using the primers Ccr1l1 F5 (ccgcttaagccaccatggaacaaaaactcatctcagaagaggatctgg) and Ccr1l1 R2; a synthetic ghCcr1l1 was obtained from GeneArt (Life Technologies) and amplified using the primers ghCcr1l1 F2 (ccgcttaagccaccatggaacaaaaactcatctcagaagaggatctggagatttcagccatcacag) and ghCcr1l1 R1 (cggtctagattataagccagcagaaagc); and mouse Ccr1 was amplified by PCR from an existing plasmid in our laboratory using the primers Ccr1 F5 (ggccttaagccaccatggaacaaaaactcatctcagaagaggatctggagatttcagatttcacag) and Ccr1 R1 (ccgtctagattagaagccagcagagagctc). PCR fragments were cloned in a pcDNA3.1 plasmid, and the resulting plasmids were termed pAT20 (mouse Ccr1l1), pAT17 (golden hamster Ccr1l1), and pAT15 (mouse Ccr1). Plasmids for the expression of the Ccr1 Y18F and Ccr1l1 F19Y mutants were obtained by site-directed mutagenesis using the QuikChange Lightning kit (Agilent, Santa Clara, CA) with the primer pairs Y18F F1/R1 (gtggagtccccaaagtcaaattctgtagttgtgggg/ccccacaactacagaatttgactttggggactccac) and F19Y F1/R1 (ctgaagcataagaatccagacatatagtcattctttggcaactgtgttg/caacacagttgccaagaatgactatatgtctggattcttatgcttcag) on the templates pAT15 and pAT20, respectively. The correct incorporation of the desired mutations was confirmed by DNA sequencing, and the resulting plasmids were termed pAT15mut and pAT20mut, respectively.

For BRET assays, mouse Ccr1 and Ccr1l1 were cloned in a pcDNA-Rluc8 plasmid for their expression in frame with a C-terminal renilla luciferase variant 8 (Rluc8) reporter gene. A 6-amino acid linker (GGGSGG) was introduced between the receptor and the reporter gene to allow the proper folding and functioning of both domains. For this, first, Ccr1 and Ccr1l1 were amplified by PCR from pAT15 and pAT20, respectively, using the primers Rluc8pJK1/2F (ctatagggagacccaagctgggccaccatggaacaaaaactc) and Rluc8pJK1R (gtcgtacaccttggaagccatgccgccgctgccgccgccgaagccagcagagagctcatg), or Rluc8pJK1/2F and Rluc8pJK2R (gtcgtacaccttggaagccatgccgccgctgccgccgcctaagtcggcagaggtctc), respectively. Then, PCR fragments were cloned into a pcDNA-Rluc8.6-535 plasmid using the NEBuilder HiFi DNA Assembly Kit (New England Biolabs, Ipswich, MA) following the manufacturer's instructions. pcDNA-Rluc8.6-535 was a gift from Sanjiv Sam Gambhir (Addgene plasmid #87125). The resulting plasmids were termed pJK1 (Ccr1-Rluc8) and pJK2 (Ccr1l1-Rluc8). The sequence of all constructs was confirmed by DNA sequencing.

### Transient expression of Ccr1l1 in HEK293 cells

To confirm that the Ccr1l1 ORF encodes a plasma membrane protein, HEK293 cells were transiently transfected with the HA-tagged Ccr1l1-expressing plasmids pNT1 and pNT2. For this, cells plated in six-well plates at one million cells/well were transfected using Fugene HD (Promega) with 2 μg of pNT1, pNT2, or pcDNA3.1 as control. Ccr1l1 expression was analyzed 48 h after transfection by FACS or fluorescence microscopy. For FACS, cells were collected with 1 mM EDTA in PBS and washed twice with PBS-staining buffer (1X PBS, 1% BSA, and 1% FBS). Then, 400,000 cells were stained with Zombie Violet viability dye (Biolegend) for 10 min at room temperature and a rabbit anti-HA tag mAb (Cell Signaling Technology, Danvers, MA) on ice for 20 min followed by an anti-rabbit F(ab′)_2_ fragment conjugated with AlexaFluor 488 (Cell Signaling Technology). Both antibodies were used at a 1:800 dilution. In total, 20,000 events were collected in a BD Fortessa bioanalyzer and analyzed using FlowJo (both from BD Biosciences). Only single cells (identified by SSC-H *versus* SSC-A gating) negative for the viability staining (live cells) were included in the analysis. For fluorescence microscopy analysis, cells were plated as indicated above in six-well plates containing microscopy coverslips. After transfection, coverslips were fixed with 2% methanol-free formaldehyde (Sigma) in PBS for 15 min at room temperature, then stained with 5 μg/ml of an anti-HA tag mAb (Sigma) and an anti-mouse-AlexaFluor488 secondary antibody (Life Technologies) in PBS containing 5% FBS with or without 0.1% of Triton X-100. Samples were mounted using Prolong Gold mountant with DAPI and imaged in an AxioVert 200 inverted fluorescence microscope (Zeiss).

### Generation of chemokine receptor-expressing stable lines

L1.2 cells were used for the generation of stable lines for the expression of Ccr1 and Ccr1l1. For this, two million cells were transfected with 2 μg of pAT15, pAT15mut, pAT20, pAT20mut or pAT17 using SG Cell Line kit in a 4D-Nucleofector X (both from Lonza) following the manufacturer's instructions. Two days after transfection, cells were plated in 24-well plates in pools containing 2400 cells/well in selection media (RPMI-Glutamax, 10% FBS, 55 mM 2-mercaptoethanol, 0.1 mM nonessential amino acids, 1 mM sodium pyruvate, and 1 mg/ml geneticin). Media was replaced with fresh selection media on day 7, and cells were cultured for seven additional days. Then, surviving pools were screened for the expression of myc-tagged receptors by FACS. For this, Fc receptors were blocked using mouse TruStain FcX (Biolegend), and receptor surface expression was detected by staining with a PE-conjugated anti-Myc antibody (clone 9B11; Cell Signaling Technology) in PBS-staining buffer (1X PBS, 1% FBS and 1% BSA). Data were acquired in a BD LSR II and analyzed using FlowJo (both from BD Biosciences). Pools with the highest expression levels were further selected by single cell limiting dilution in selection media. Clones were screened for receptor expression by FACS as explained before. Selected clones were expanded and frozen in geneticin-free selection media. For experiments, clones were grown and maintained in selection media containing 1 mg/ml geneticin at all times.

### Internalization assays

L1.2 stable cell lines expressing myc-tagged chemokine receptors were seeded into a 96-well plate at 1 × 10^6^ cells/well in internalization buffer (RPMI-Glutamax supplemented with 0.5% BSA and 10 mM HEPES). Cells were incubated in the presence or absence of 100 nM chemokine in internalization buffer for 45 min at 37 °C and 5% CO_2_. Subsequently, cell surface receptor levels were analyzed by FACS as explained above.

### Bioluminescence resonance energy transfer (BRET)

The capacity of Ccr1 and Ccr1l1 to recruit β-arrestin in a ligand-dependent or -independent way was analyzed by BRET. For this, 293T cells were transiently cotransfected using Fugene HD (Promega) with a beta-arrestin2 mYFP plasmid and pJK1 (Ccr1-Rluc8), pJK2 (Ccr1l1-Rluc8) or empty pcDNA-Rluc8 as control. Beta-arrestin2 mYFP plasmid was a gift from Robert Lefkowitz (Addgene plasmid # 36917). Twenty-four hours after transfection, cells were collected, plated in clear-bottom 96-well white plates at 10^5^ cells/well, and cultured for an additional 24 h. Then, media was removed and 30 μl/well of assay buffer (1X PBS, 0.1% BSA and 0.5 mM MgCl) was carefully added on top of cells. Subsequently, coelenterazine h (5 μM final concentration) and 100 nM of the indicated chemokines in assay buffer or buffer alone were added after obscuring the bottom of the plate with opaque adhesive, and the luminescence (480 nm) and fluorescence (540 nm) signals were recorded over time (up to 80 min) using a Mithras LB 940 Multimode Microplate Reader (Berthold Technologies). BRET was calculated as the YFP/Rluc8 emission ratio. The BRET signal recorded in wells transfected only with an Rluc8-containing plasmid was subtracted from the BRET in sample wells to calculate the net BRET in each condition.

### Calcium flux assays

L1.2 stable cell lines or BMDE were seeded into clear-bottom 96-well black plates at 400,000 or 200,000 cells/well in 100 μl of media, respectively. Cells were incubated for 20–30 min at 37 °C and then incubated with 100 μl of FLIPR Calcium 6 Assay Kit (Molecular Devices) for 2 h at 37 °C. Chemokine dilutions were prepared at ninefold of the indicated final concentration in a 1:1 vol:vol mix of cell culture media and calcium assay buffer (HBSS supplemented with 20 mM HEPES). Then, 25 μl/well of chemokine solution or buffer alone was added using the robotic pipettor of a FlexStation 3 microplate reader (Molecular Devices), and the relative fluorescence signal was recorded (em: 485 nm; ex: 525 nm) for 180 s.

### Chemotaxis assays

Chemotaxis of BMDE and L1.2 cells was tested using ChemoTx 96-well plates (NeuroProbe) with 3 or 5 μm pore size membranes, respectively. To enhance the expression of the transfected receptors, L1.2 stable lines were incubated with 5 μM butyric acid (Sigma) for 18 h before the experiment. Recombinant chemokines at the indicated concentrations in assay buffer (RPMI-GlutaMax supplemented with 0.5% BSA and 10 mM HEPES) were placed in the bottom wells. In total, 200,000 cells in assay buffer were placed on the top membrane and allowed to migrate for 4 h or 30 min at 37 °C for L1.2 cells and BMDE, respectively. Then, the top membrane was removed, and the total number of migrated cells was calculated by interpolation in a cell standard curve after incubation with Cell Titer Aqueous One Solution Kit (Promega) and determination of the absorbance at 490 nm in a FlexStation 3 microplate reader (Molecular Devices).

### Computational analyses

Mouse Ccr1l1 (NCBI reference sequence number NP_031744.3) protein was used as query in BLAST-P and I-TASSER analyses. No restrictions or template sequences were introduced in I-TASSER, which selected human CCR5 as the best structure existing in the databases to generate a homology model of Ccr1l1. Images of the predicted three-dimensional structure of Ccr1l1 were generated using Chimera. All the sequence alignments and phylogenetic analyses were generated and edited using the sequence analysis and tree/dendrogram building tools in the Geneious software platform.

## Data availability

All data are contained within the article.

## Conflict of interest

The authors declare that they have no conflicts of interest with the contents of this article.
